# Mitofusin 2 Deficiency Causes Pro-Inflammatory Effects in Human Primary Macrophages

**DOI:** 10.3389/fimmu.2021.723683

**Published:** 2021-08-12

**Authors:** Vera Khodzhaeva, Yannick Schreiber, Gerd Geisslinger, Ralf P. Brandes, Bernhard Brüne, Dmitry Namgaladze

**Affiliations:** ^1^Institute of Biochemistry I, Faculty of Medicine, Goethe-University Frankfurt, Frankfurt, Germany; ^2^Fraunhofer Institute for Translational Medicine and Pharmacology ITMP, Frankfurt, Germany; ^3^Institute of Clinical Pharmacology, Pharmazentrum Frankfurt/ZAFES, University Hospital, Goethe-University Frankfurt, Frankfurt, Germany; ^4^Institute for Cardiovascular Physiology, Goethe-University Frankfurt, Frankfurt, Germany; ^5^German Cancer Consortium (DKTK), Partner Site Frankfurt, Frankfurt, Germany; ^6^Frankfurt Cancer Institute, Goethe-University Frankfurt, Frankfurt, Germany

**Keywords:** macrophages, mitochondria, zymosan, inflammation, mitochondrial dynamics, endoplasmic reticulum

## Abstract

Mitofusin 2 (MFN2) is a mitochondrial outer membrane GTPase, which modulates mitochondrial fusion and affects the interaction between endoplasmic reticulum and mitochondria. Here, we explored how MFN2 influences mitochondrial functions and inflammatory responses towards zymosan in primary human macrophages. A knockdown of MFN2 by small interfering RNA decreased mitochondrial respiration without attenuating mitochondrial membrane potential and reduced interactions between endoplasmic reticulum and mitochondria. A MFN2 deficiency potentiated zymosan-elicited inflammatory responses of human primary macrophages, such as expression and secretion of pro-inflammatory cytokines interleukin-1β, -6, -8 and tumor necrosis factor α, as well as induction of cyclooxygenase 2 and prostaglandin E_2_ synthesis. MFN2 silencing also increased zymosan-induced nuclear factor kappa-light-chain-enhancer of activated B cells and mitogen-activated protein kinases inflammatory signal transduction, without affecting mitochondrial reactive oxygen species production. Mechanistic studies revealed that MFN2 deficiency enhanced the toll-like receptor 2-dependent branch of zymosan-triggered responses upstream of inhibitor of κB kinase. This was associated with elevated, cytosolic expression of interleukin-1 receptor-associated kinase 4 in MFN2-deficient cells. Our data suggest pro-inflammatory effects of MFN2 deficiency in human macrophages.

## Introduction

Mitochondria are intracellular organelles involved, in addition to their classic role in production of ATP and biosynthetic intermediates (1, 2) in diverse cellular functions such as apoptosis (3), calcium homeostasis (4), heme biosynthesis (5), as well as antiviral immunity (6, 7). Mitochondria are highly dynamic organelles undergoing constant changes in their morphology through fusion and fission of mitochondrial networks (8). Mitochondrial outer membrane fusion is mediated by two mitofusin (MFN) isoforms MFN1 and MFN2, whereas optic atrophy 1 protein is critical for the fusion of inner mitochondrial membranes (9). MFN1 and MFN2 show 77% similarity, with N-terminal GTPase domain and a single membrane-spanning segment, and can substitute each other to mediate mitochondrial fusion (10). However, they differ in catalytic GTPase activity, and ablation of MFN1 or MFN2 results in different fragmentation phenotypes (10). Moreover, MFN2 is required for the interactions between mitochondria and the endoplasmic reticulum (ER), thereby controlling calcium and phospholipid homeostasis (11–14).

Dysfunction of MFN2 contributes to metabolic disorders, cardiovascular, neurodegenerative, and neuromuscular diseases (15). For instance, mutations in MFN2 are associated with Charcot-Marie-Tooth neuropathy type 2A and optic atrophy (16–18). Age-related MFN2 deficiency increases sarcopenia (19). In addition, expression of MFN2 is reduced in obesity and type II diabetes patients (20–22). MFN2 down-regulation increases ROS generation, and activates JNK signaling pathway, causing the formation of lipid intermediates that result in insulin resistance in liver and skeletal muscle (20, 21, 23). Mechanistically, MFN2 deficiency suppresses mitophagy (19, 24, 25), which causes the accumulation of damaged mitochondria with various deleterious consequences, such as oxidative stress, impaired mitochondrial metabolism, and cell death. In addition, MFN2 inhibits mitochondrial antiviral signaling *via* interaction with mitochondrial antiviral signaling protein (26).

Macrophages are essential mediators of innate immunity with key roles in inflammatory responses (27). A few studies addressed the role of MFN2 in macrophages (28–30). Thus, MFN2 enhanced cellular cholesterol transporter expression in THP-1-differentiated human macrophages by up-regulating peroxisome proliferator-activated receptor-γ (28). MFN2 was also required for NLRP3 inflammasome activation in bone marrow-derived mouse macrophages after viral infection (29). Recent study of myeloid-specific MFN2 knockout mice revealed impaired mitochondrial reactive oxygen species (mROS) production and associated defects of pro-inflammatory activation in MFN2-deficient macrophages (30). Whether MFN2 similarly regulates inflammatory responses of human macrophages is currently unclear.

In this study we investigated the effect of MFN2 deficiency induced by small interfering RNA (siRNA) on mitochondrial functions and inflammatory responses of primary human macrophages. We demonstrate that MFN2 deficiency reduces mitochondrial respiration and attenuates interactions between endoplasmic reticulum and mitochondria. Unexpectedly, MFN2 silencing increased zymosan-induced inflammatory response *via* toll-like receptor 2 (TLR2) activation. Mechanistically, a MFN2 knockdown enhanced nuclear factor kappa-light-chain-enhancer of activated B cells (NF-κB) signaling associated with elevated cytosolic expression of interleukin-1 receptor-associated kinase 4 (IRAK4) signaling complex.

## Materials and Methods

### Primary Human Macrophage Isolation, Differentiation, and Treatment

Human peripheral blood mononuclear cells (PBMCs) were isolated from commercially available buffy coats from anonymous donors (DRK-Blutspendedienst Baden-Württemberg-Hessen, Institut für Transfusionsmedizin und Immunhämatologie, Frankfurt, Germany) using Ficoll (Biochrom, Berlin, Germany) density centrifugation. Monocytes were isolated from PBMCs using positive selection with CD14 monoclonal antibody-coupled magnetic beads (MACS Miltenyi Biotec, Bergisch Gladbach, Germany) following the manufacturer’s protocol. CD14^+^ monocytes were differentiated in macrophage serum-free medium (ThermoFisher Scientific, Waltham, MA, USA) supplemented with 100 U/mL penicillin, 100 μg/mL streptomycin, and 50 ng/mL macrophage colony-stimulating factor (M-CSF) (Immunotools, Friesoythe, Germany) to support the survival and differentiation for 7 days. Cells were grown under standard conditions (5% CO_2_, 37°C, water-saturated atmosphere) at 10^6^/well in 6-well plates for RNA and protein analyses. When indicated, macrophages were treated with 50 µg/ml zymosan A (S. cerevisiae) (Sigma-Aldrich), 10 ng/ml Pam2CSK4 (PAM2, InvivoGen) or 100 ng/mL lipopolysaccharide (LPS, Sigma-Aldrich) to induce pro-inflammatory activation of macrophages, 5 µM Src I (Sigma-Aldrich), 10 and 50 µM MitoTEMPO (Cayman). To block tyrosine phosphatases when checking for spleen tyrosine kinase (Syk) and Src kinase activation, cells were pre-incubated with sodium orthovanadate Na_3_VO_4_ for 30 min followed by stimulation with zymosan for 30 min.

### siRNA Transfections

Control siRNA and siRNAs targeting human MFN2 and MFN1 (siGENOME human SMARTpool, Horizon Discovery Biosciences, Cambridge, United Kingdom) were transfected into primary human macrophages at a final concentration of 50 nM using HiPerFect transfection reagent (Qiagen, Hilden, Germany) according to the manufacturer’s recommendations. Each knockdown was routinely confirmed by quantitative polymerase chain reaction (q-PCR) and Western blot for each experiment.

### RNA Isolation and q-PCR

Total RNA was isolated using TRIzol reagent (Life Technologies, Carlsbad, CA, USA) followed by reverse transcription using Maxima first-strand cDNA synthesis kit (ThermoFisher Scientific). q-PCR assays were performed with PowerUp SYBR Green Master Mix (Applied Biosystems, Foster City, CA, USA) using Quant Studio Real Time PCR System (Applied Biosystems). Relative transcript amounts were quantified using Δct method with β-microglobulin (βMG) as a housekeeping gene and normalized to the untreated or agonist-treated controls. Primer sequences are listed in **Table 1**.

**Table 1:** Primers used for q-PCR analyses.

Name	Sequence
IL1β forward	TTCGACACATGGGATAACGA
IL1β reverse	TCTTTAACGCAGGACAG
IL10 forward	GACTTTAAGGGTTACCTGGGTTG
IL10 reverse	TCACATGCGCCTTGATGTCTG
IL6 forward	TCAATGAGGAGACTTGCCTGGTGA
IL6 reverse	TACTCATCTGCACAGCTCTGGCTT
IL8 forward	AGCCTTCCTGATTTCTGCAGCTCT
IL8 reverse	AATTTCTGTGTTGGCGCAGTGTGG
IRAK1 forward	GCACCCACAACTTCTCGGAG
IRAK1 reverse	CACCGTGTTCCTCATCACCG
IRAK4 forward	CCTGACTCCTCAAGTCCAGAA
IRAK4 reverse	ACAGAAATGGGTCGTTCATCAAA
MFN1 forward	ACGCCTTAGTGCTTCAGACC
MFN1 reverse	GCATTATCTGGCGTTGCTGG
MFN2 forward	CTCTCGATGCAACTCTATCGTC
MFN2 reverse	TCCTGTACGTGTCTTCAAGGAA
TLR2 forward	GGCGTTCTCTCAGGTGACTG
TLR2 reverse	TCCAGTGCTTCAACCCACAA
TLR6 forward	TCTTCCTCCTGAAAGCAGAAGT
TLR6 reverse	TTCCGTCGGAGAACTGGATTC
TNFα forward	GAGGCCAAGCCCTGGTATG
TNFα reverse	CGGGCCGATTGATCTCAGC

### Western Blot Analysis

Cells were harvested in lysis buffer [50 mM Tris-HCl, pH 8, 150 mM NaCl, 5 mM EDTA, 10 mM NaF, 1mM Na_3_VO_4_, 0.5% NP-40, 1 mM phenylmethylsulfonyl fluoride (PMSF), protease inhibitor cocktail (11697498001, Sigma-Aldrich), and PhosStop (4906845001, Sigma-Aldrich)]. Protein lysates were sonicated and centrifuged at 12000 g for 10 min at 4°C. Supernatants were heat-denatured at 95°C, separated on SDS-PAGE gels, and transferred onto nitrocellulose membranes. We used primary antibodies as indicated in **Table 2**. Following incubations with primary antibodies, the membranes were incubated with IRDye 680 or IRDye 800-coupled secondary antibodies (LI-COR Biosciences, Bad Homburg, Germany). Proteins were visualized and densitometrically analyzed with the Odyssey imaging system (LI-COR Biosciences).

**Table 2:** Antibodies used for western blot analyses.

Name	Source	Catalogue number
ACLY	Proteintech	15421-1-AP
COX2	Cell Signaling Technology	#12282
CPT1A	Proteintech	15184-1-AP
IκBα	Cell Signaling Technology	#4814
IKKβ	Cell Signaling Technology	#8943
IRAK1	Cell Signaling Technology	#4359
IRAK4	Abcam	ab119942
JNK	Cell Signaling Technology	#9252
MFN1	R&D	AF7880-SP
MFN2	Proteintech	12186-1-AP
nucleolin	Santa Cruz Biotechnology	sc-13057
p38-MAPK	Cell Signaling Technology	#9212
p44/42 MAPK (ERK1/2)	Cell Signaling Technology	#4696
phospho-IκBα (S32)	Cell Signaling Technology	#2859
phospho-IKKα/β (S176/180)	Cell Signaling Technology	#2697
phospho-IRAK4 (T345/S346)	Cell Signaling Technology	#11927
phospho-JNK (T183/Y185)	Cell Signaling Technology	#4668
phospho-p38-MAPK (T180/Y182)	Cell Signaling Technology	#4511
phospho-p44/42 MAPK (ERK 1/2) (T202/Y204)	Cell Signaling Technology	#9101
phospho-Src (Y416)	Cell Signaling Technology	#2101
phospho-Syk (Y525-526)	Cell Signaling Technology	#2711
phospho-TAK1 (T184)	Cell Signaling Technology	#4537
Src	Cell Signaling Technology	#2109
Syk	Cell Signaling Technology	#2712
TAK1	Cell Signaling Technology	#4505
TRAF6	Cell Signaling Technology	#8028

### Cytometric Bead Array

To determine TNFα, IL-6, and IL-1β levels in macrophage supernatants Cytometric Bead Array Flex Sets (BD Biosciences) were used according to manufacturer’s instructions. Macrophages seeded at 10^6^/well in 6-well plates were incubated with 50 µg/ml zymosan for 24 h prior to analysis. Samples were measured using a FACSymphony A5 flow cytometer (BD Biosciences), and data were analyzed using BD Biosciences FCAP software (V3.0).

### Prostanoid Analysis

Prostanoid analysis was carried out as previously described (31). Macrophages were cultured at 10^6^/well in 6-well plates. Briefly, 200 μL of supernatant was spiked with the isotopically labeled internal standards and extracted using ethyl acetate. The chromatographic separation of the analytes was carried out using a Synergi Hydro-RP column (150 × 2 mm, 4 μm particle size and 80 Å pore size; Phenomenex, Aschaffenburg, Germany) under gradient conditions. Water and acetonitrile, both containing 0.0025% formic acid, were used as mobile phases and sample run time was 16 min. The MS/MS system consisted of a hybrid triple quadrupole-ion trap mass spectrometer QTrap 6500+ (Sciex, Darmstadt, Germany) equipped with a Turbo-V-source operating in the negative electrospray ionization mode. Analysis was done in the Multiple Reaction Monitoring mode. Data were acquired using Analyst Software V 1.6.3 and quantified with MultiQuant Software V 3.0.2 (both Sciex), using the internal standard method (isotope dilution mass spectrometry).

### Mitochondrial Respiration

A Seahorse XF96 extracellular flux analyzer (Seahorse Bioscience, Agilent, Santa Clara, CA, USA) was used to determine the metabolic profile of cells. Macrophages were plated in Seahorse 96-well cell culture plates at 5x10^4^ cells/well one day before the assay and equilibrated for 1 h in Krebs Henseleit buffer (111 mM NaCl, 4.7 mM KCl, 1.25 mM CaCl_2_, 2 mM MgSO_4_, 1.2 mM NaH_2_PO_4_, pH 7.4) supplemented with 11 mM L-glucose and 2 mM L-glutamine. Cells were treated with 2.5 µM oligomycin (Sigma-Aldrich), 1 µM carbonyl cyanide 3-chlorophenylhydrazone (CCCP, Sigma-Aldrich), 1 µg/ml antimycin (Sigma-Aldrich) and 1 µM rotenone (Sigma-Aldrich) as indicated. Oxygen consumption rates (OCR) and extracellular acidification rates (ECAR) were monitored in real time after injection of each compound.

### Microscopy

Human macrophages were seeded at 1x10^5^ cells/well in μ-Slide 8-well chambers (80826, Ibidi, Martinsried, Germany) and stained with 0.5 µM MitoTracker Green (Thermo Fisher Scientific) for 15 min at 37°C before live imaging. The nuclei were stained using 2 µM Hoechst (Thermo Fisher Scientific). Imaging was performed using confocal microscope (LSM800, Carl Zeiss, Oberkochen, Germany), driven by Zen 2009 software (Carl Zeiss). Scoring of mitochondrial network morphology was performed by ImageJ software. Form factor (FF) was used to determine mitochondrial shape (32). FF [(perimeter2)/(4π•surface area)] reflects combined measure of length and degree of branching. All quantifications were done in more than 50 cells per experiment and n≥3 of experiments.

### Proximity Ligation Assay

Voltage-dependent anion channel 1 (VDAC1) and inositol 1,4,5-trisphosphate receptor type1 (IP3R1) proximity were measured by *in situ* proximity ligation assay (PLA) (Duolink II Fluorescence, OLink, Upsalla, Sweden) to detect, visualize and quantify ER-mitochondria interactions (33). Briefly, macrophages were plated at 1x10^5^ cells/well in μ-Slide 8-well chambers (80826, Ibidi), fixed with ROTI histofix 4% (Carl Roth, Karlsruhe, Germany), permeabilized with Triton-X 100 (0.05% in phosphate-buffered saline (PBS)), blocked and incubated overnight with antibodies against VDAC1 (ab14734, Abcam) and IP3R1 (07-1213, Millipore). After washing, samples were incubated with the respective PLA-probes for one hour (37°C), washed and ligated for 30min (37°C). After an additional washing, amplification with polymerase was allowed for 100 min (37°C). The nuclei were stained using 1µg/ml DAPI (Sigma-Aldrich). Images were acquired by confocal microscope (LSM 800, Carl Zeiss). Protein-protein interactions are represented as individual fluorescent dots. 10 fields of about 20 cells per condition for each experiment were acquired, and quantification of detected PLA spots per cell was then analyzed using Image J software.

### Phagocytosis Assay

Primary macrophages plated at 10^5^/well in 24-well plates were incubated with Alexa Fluor 594 labeled zymosan particles (10 µg/ml) (Z23374, Thermo Fisher Scientific) for 30 min and 60 min. To obtain homogenous suspension zymosan was sonicated and also vortexed. Then cells were washed with PBS to remove all uninternalized zymosan particles. Fluorescence was measured by FACSymphony A5 flow cytometer (BD Biosciences).

### Measurements of Mitochondrial Membrane Potential

The mitochondrial membrane potential was measured by loading macrophages plated at 10^5^/well in 24-well plates with 5 µM 5,5′,6,6′-tetrachloro-1,1′,3,3′-tetraethyl benzimidazolcarbocyanine iodide (JC-1, BioVision, Milpitas, CA, USA) for 30 minutes at 37°C after treatments with zymosan. Fluorescence was measured by FACSymphony A5 flow cytometer (BD Biosciences).

### H_2_O_2_ Measurement With Amplex Red

Macrophages were plated at 50x10^4^/well in 96-well black plates. Hydrogen peroxide or total cellular ROS was measured using Amplex Red hydrogen peroxide/peroxidase assay kit (Thermo Fisher Scientific) upon zymosan stimulation for 30 min following the manufacturer’s protocol.

### Mitochondrial ROS Detection

mROS generation in macrophages was detected by MitoSOX (Thermo Fisher Scientific). Macrophages plated at 10^5^/well in 24-well plates were incubated with 5 μM MitoSOX for 30 min at 37°C after treatments with zymosan. Fluorescence was analyzed using FACSymphony A5 flow cytometer (BD Biosciences).

### Cellular Fractionation and Mitochondria Isolation

Macrophages cultured at 10^6^/well in 6-well plates were rinsed with cold PBS, and then harvested in cold mitochondria buffer (250 mM sucrose, 1 mM EDTA, 20 mM Tris-HCl, protease and phosphatase inhibitor cocktails, pH 7.5). Cell suspensions were homogenized with a Teflon pestle followed by centrifugation at 1000 g for 10 min 2 times to pellet nuclei and unbroken cells. The supernatant was transferred to a fresh tube and spun at 15000 g for 10 min to pellet mitochondria, the resulting cytosolic supernatant was centrifuged one more time to avoid mitochondrial contamination. Mitochondria pellet was re-suspended in a fresh buffer and centrifuged for a second time at 15000 g for 10 min. Fraction purity was confirmed by blotting for ACLY (cytosol) and CPT1A (mitochondria).

### Statistical Analysis

Graphical data are presented as means ± SEM of at least three independent experiments using human primary macrophages derived from different individual donors. Statistical significance was calculated using one-way analysis of variance (ANOVA) with Dunnet’s or Sidak’s multiple comparisons test or Student unpaired, two-tailed t-test using Prism 8 software (GraphPad, La Jolla, CA, USA). Differences were considered significant when p < 0.05 (*p < 0.05; **p < 0.01; ***p < 0.001; ****p < 0.0001; ns = not significant).

## Results

### Mitochondria in MFN2-Silenced Human Primary Macrophages

**Figure 1:**
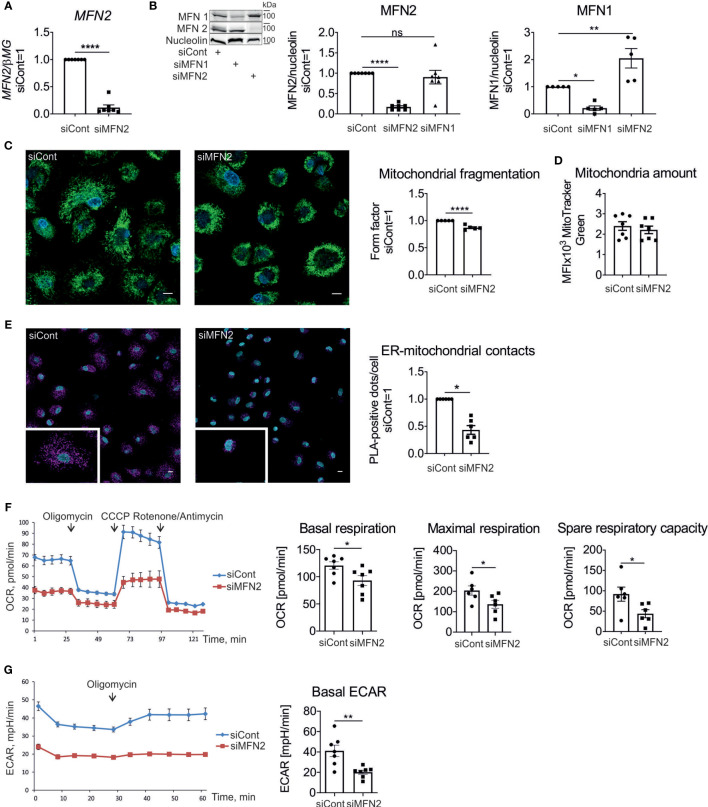
Characterization of mitochondria in MFN2-silenced human macrophages. **(A)** MFN2 mRNA expression in human primary macrophages transfected with non-targeting control siRNA (siCont) or MFN2 siRNA (siMFN2). **(B)** Western blot analysis and quantification of MFN2 and MFN1 proteins in macrophages transfected with control (siCont), MFN2 (siMFN2), and MFN1 (siMFN1) siRNAs. **(C)** Representative images of macrophages transfected with siCont or siMFN2 and stained with MitoTracker Green (mitochondria) and Hoechst (nuclei). Quantification of the mitochondrial fragmentation using form factor parameter. Scale bars = 10 μm. **(D)** Total amount of mitochondria calculated by staining with MitoTracker Green. **(E)** Representative images of the interactions between VDAC1 and IP3R1 (pink dots) analyzed by proximity ligation assay in macrophages transfected with siCont or siMFN2. Nuclei are stained with DAPI (blue). Scale bars = 10 μm. The number of VDAC1/IP3R1 interactions (ER-mitochondrial contacts) per cell was analyzed using Image J software. **(F)** Seahorse profile of oxygen consumption rates (OCR) in macrophages transfected with siCont or siMFN2 following treatments with oligomycin, CCCP, rotenone, and antimycin. Bar charts showing basal respiration, maximal respiration, and spare respiratory capacity of control and MFN2-silenced macrophages. **(G)** Extracellular acidification rate (ECAR) under basal conditions and after the addition of oligomycin. All results are shown as means ± SEM of at least three independent experiments compared using one-way analysis of variance (ANOVA) with Dunnet’s multiple comparisons test or Student unpaired, two-tailed t-test; *p < 0.05; **p < 0.01; ****p < 0.0001; ns , not significant. Photographs are representative of five **(C)** and six **(E)** independent experiments.

To examine MFN2 function in human primary macrophages, we performed a MFN2 knockdown using siRNA. This reduced MFN2 mRNA (**Figure 1A**) and protein (**Figure 1B**) by over 90%.

MFN1 and MFN2 are homologues proteins with redundant roles in mitochondrial fusion (9). To determine how silencing of MFN1 and MFN2 affects its homologue’s expression we assessed protein levels of MFN2 and MFN1 after silencing. MFN1 expression increased in MFN2 knockdown macrophages, while there was no change in the MFN2 amount after a MFN1 knockdown (**Figure 1B**). These findings indicate that MFN1 is compensatorily upregulated in MFN2-deficient macrophages. Analyzing mitochondrial morphology in macrophages stained with the mitochondrial dye Mitotracker Green revealed only a minor fragmentation of mitochondrial networks (**Figure 1C**). Quantification of mitochondrial connectivity, using mitochondrial form factor (34), showed only a small reduction. Total amount of mitochondria per cell, as determined by flow cytometry of Mitotracker Green-stained macrophages, was unaltered (**Figure 1D**).

Next, we investigated the impact of MFN2 silencing on ER-mitochondrial contacts in human macrophages. Using the *in situ* PLA we analyzed the proximity between VDAC1 on the outer mitochondrial membrane and IP3R1 on the ER membrane (33). Interactions between VDAC1 and IP3R1 were identified as red dots by confocal fluorescence microscopy and quantified with ImageJ software. Comparing cells transfected with control and MFN2 siRNAs, we found that the number of PLA dots per cell decreased after MFN2 silencing, suggesting reduced ER-mitochondrial contacts in MFN2 knockdown macrophages (**Figure 1E**).

To examine the influence of MFN2 on mitochondrial metabolism of human macrophages we performed Seahorse extracellular flux assay. Following the basal OCR, addition of the ATP synthase inhibitor oligomycin revealed ATP-linked respiration, whereas adding the mitochondrial uncoupler CCCP allowed to determine maximal respiration and spare respiratory capacity. Injecting a mixture of respiratory complex I inhibitor rotenone and complex III inhibitor antimycin A almost completely suppressed oxygen consumption, confirming the mitochondrial dependence of OCR. MFN2 knockdown in macrophages showed decreased basal OCR, maximal respiration and spare respiratory capacity in comparison with control siRNA-transfected cells (**Figure 1F**). Measuring the ECAR showed decreased basal ECAR in MFN2-silenced macrophages (**Figure 1G**). These results indicate that MFN2 knockdown reduces mitochondrial activity in macrophages without causing switch to glycolysis.

### MFN2 Deficiency Enhances Macrophage Pro-Inflammatory Responses to Zymosan

**Figure 2:**
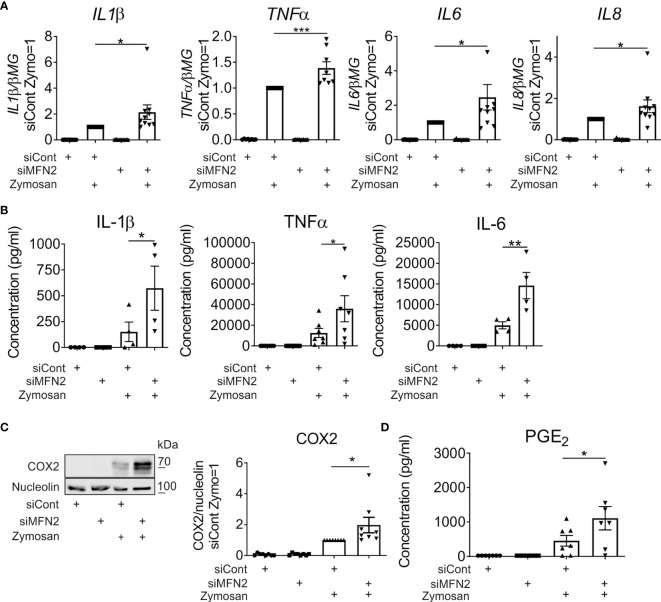
MFN2-silenced macrophages show increased pro-inflammatory responses to zymosan. **(A)** mRNA expression of indicated genes in macrophages transfected with control and MFN2 siRNAs and treated with 50 µg/ml zymosan for 3 h **(B)** TNFα, IL-6, and IL-1β secretion to supernatants of control and MFN2-silenced macrophages. Macrophages were incubated with 50 µg/ml zymosan for 24 h prior to analysis. **(C)** Western blot analysis and quantification of cyclooxygenase-2 (COX2) protein in control and MFN2-silenced macrophages after 24 h incubation with 50 µg/ml zymosan. **(D)** Prostaglandin E_2_ (PGE_2_) release into culture medium of control and MFN2-silenced macrophages after 24 h treatment with 50 µg/ml zymosan. All results are shown as means ± SEM of at least three independent experiments compared using one-way analysis of variance (ANOVA) with Sidak’s multiple comparisons test or Student unpaired, two-tailed t-test; *p < 0.05; **p < 0.01; ***p < 0.001.

Zymosan, a glucan derived from yeast cell walls, is a potent activator of inflammatory cytokines and prostanoid production, both in murine and human macrophages (35–39). To investigate the impact of MFN2 silencing in zymosan-stimulated human primary macrophages we initially analyzed gene expression of pro-inflammatory cytokines 3 h after treatments with 50 µg/ml zymosan by q-PCR. Expression of interleukin (*IL) 1β*, tumor necrosis factor alpha *(TNFα), IL6* and *IL8* was elevated in zymosan-stimulated MFN2 knockdown macrophages as compared to siRNA transfected cells (**Figure 2A**). In contrast, MFN1-silenced macrophages showed unaltered induction of most of the analyzed cytokines (**Supplementary Figure 1**). The secretion of IL1β, TNFα, and IL6 proteins was also increased upon zymosan treatment in MFN2-silenced cells (**Figure 2B**).

Next, we examined arachidonic acid metabolism upon MFN2 depletion. Zymosan potently induced protein expression of cyclooxygenase-2 (COX2), the key enzyme responsible for prostanoid generation in inflammatory macrophages. COX2 protein levels were elevated in MFN2-silenced cells as compared to control siRNA-transfected macrophages 24 h after incubation with zymosan (**Figure 2C**). To evaluate COX2-dependent prostanoid generation we measured prostaglandin E_2_ (PGE_2_) release into the culture medium after 24 h. In analogy with COX2 expression, PGE_2_ synthesis also increased in MFN2-inactivated macrophages (**Figure 2D**). These results suggest that MFN2 increases zymosan-induced inflammatory responses of human primary macrophages.

**Figure 3:**
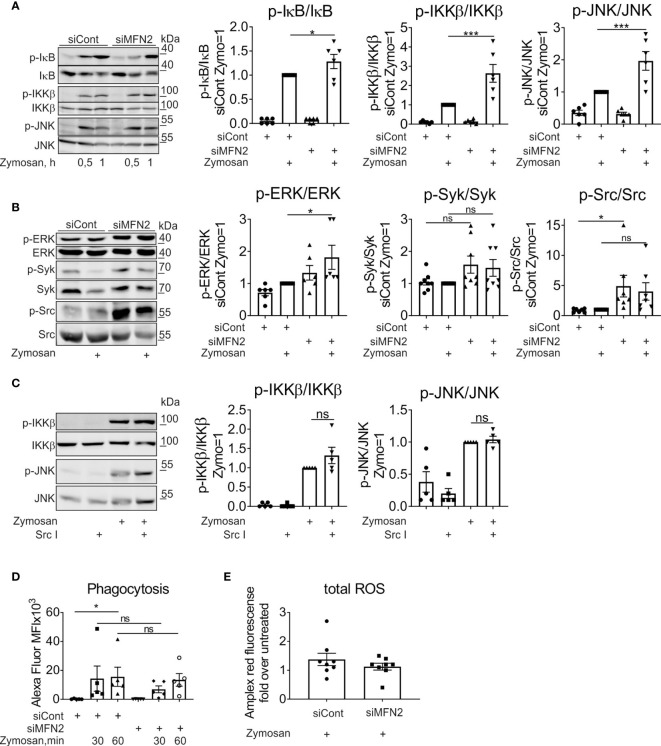
MFN2 silencing enhances NF‐κB and MAPK signal transduction pathways in zymosan-treated macrophages. **(A)** Western blot analysis of IκB, IKKβ, and JNK phosphorylation in macrophages transfected with control and MFN2 siRNAs and treated with 50 µg/ml zymosan for 0.5 or 1 h Quantification is shown only for 1 h **(B)** Western blot analysis and quantification of ERK, Syk, and Src phosphorylation in macrophages transfected with control and MFN2 siRNAs and stimulated with 50 µg/ml zymosan for 30 min. **(C)** Western blot analysis and quantification of IKKβ and JNK phosphorylation in macrophages pre-incubated for 30 min with Src inhibitor (Src I, 5 µM) followed by zymosan (50 µg/ml) stimulation for 30 min. **(D)** Median fluorescence intensity (MFI) of macrophages transfected with control or MFN2 siRNAs following phagocytosis of Alexa Fluor 594-labeled zymosan particles (10 µg/ml) for 30 or 60 min. **(E)** Total cellular ROS in zymosan-treated (50 µg/ml, 30 min) siCont or siMFN2-transfected macrophages measured using Amplex Red. All results are shown as means ± SEM of at least three independent experiments compared using one-way analysis of variance (ANOVA) with Sidak’s multiple comparisons test or Student unpaired, two-tailed t-test; *p < 0.05; ***p < 0.001; ns, not significant.

The induction of pro-inflammatory cytokines by zymosan is mediated by a series of signal transduction pathways, including nuclear factor kappa-light-chain-enhancer of activated B cells (NF‐κB) signaling and different mitogen-activated protein kinases (MAPK) such as c-Jun N-terminal kinase (JNK), extracellular signal-regulated kinase (ERK), and p38 MAPKs (35, 36, 40). Furthermore, NF‐κB and members of the MAPK family contribute to induction of COX2 (41–43). Therefore, we analyzed how MFN2 deficiency affects the activity of these signaling cascades in zymosan-treated macrophages. The major regulator of NF‐κB signaling is the inhibitor of nuclear factor kappa B (IκB) kinase enzyme complex (IKK) that consists of two kinases, IKKα and IKKβ (44). IKK phosphorylates the inhibitory IκBα protein, which provokes the dissociation of IκBα from NF-κB, allowing NF-κB nuclear translocation (45). After zymosan stimulation for 30 min and 60 min phosphorylation of IKKβ and IκB, significantly increased in MFN2-silenced macrophages (**Figure 3A**). A MFN2 knockdown also enhanced activation of MAPK kinases JNK and ERK in zymosan-treated macrophages (**Figures 3A, B**). These data show that MFN2 deficiency increases zymosan-induced inflammatory signal transduction in human primary macrophages.

Zymosan particles can be recognized mainly by dectin-1 and TLRs (46, 47). Zymosan-stimulated TLRs provoke NF-κB activation and the production of inflammatory cytokines (46). Dectin-1 ligation triggers activation of spleen tyrosine kinase (Syk) and Src tyrosine kinase in murine bone marrow-derived macrophages (48) or in primary human monocytes (49). To question the impact of MFN2 deficiency on the activity of Dectin-1 signaling pathway, we analyzed Src and Syk activation in zymosan-treated human primary macrophages. Surprisingly, we could not detect any increase of Syk phosphorylation after zymosan treatment in cells transfected with control siRNA (**Figure 3B**). The MFN2 knockdown did not change the phosphorylation level of Syk neither basally nor upon zymosan stimulation (**Figure 3B**). In contrast, the MFN2 knockdown increased basal Src phosphorylation, however no significant change in Src phosphorylation after 30 min zymosan stimulation was observed (**Figure 3B**). Next, we examined whether the Src inhibitor (Src I), a specific inhibitor of Src family kinases, influences zymosan-induced IKKβ and JNK activation. We did not see any decrease of IKKβ or JNK phosphorylation in zymosan-stimulated macrophages after pretreatment with 5µM Src I for 30 min (**Figure 3C**), suggesting that Src does not play an important role in zymosan-induced inflammatory signaling.

Dectin-1 triggers phagocytosis and ROS production in murine macrophages, thereby participating in fungal killing (46). Therefore, we examined phagocytosis and total ROS production in MFN2-inactivated zymosan-stimulated macrophages. Primary macrophages were incubated with zymosan particles labeled with Alexa Fluor 594 (10 µg/ml) for 30 min and 60 min. We noticed no difference in zymosan particle phagocytosis between control and MFN2 knockdown macrophages (**Figure 3D**). Next, we evaluated total cellular ROS production using Amplex Red hydrogen peroxide/peroxidase assay kit upon zymosan stimulation for 30 min. Again, ROS generation remained unaltered in MFN2-inactivated macrophages (**Figure 3E**). Collectively, our data suggest that dectin-1 signaling plays minor roles in zymosan-induced inflammatory responses and is not affected by MFN2-silencing in human primary macrophages.

**Figure 4:**
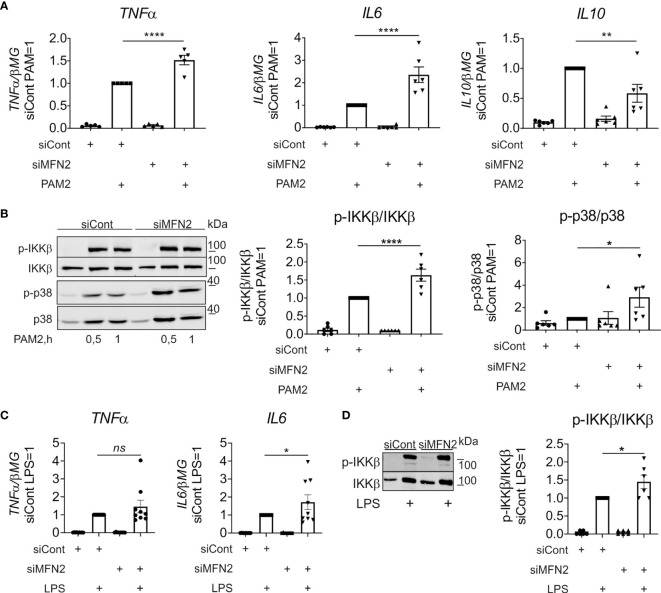
MFN2 silencing increases inflammatory responses in macrophages treated with TLR2 and TLR4 agonists. **(A)** mRNA expression of indicated genes in macrophages transfected with control and MFN2 siRNAs and treated with 10 ng/ml Pam2CSK4 (PAM2) for 3 h **(B)** Western blot analysis of IKKβ and p38 phosphorylation in macrophages transfected with control and MFN2 siRNAs and stimulated with 10 ng/ml PAM2 for 0.5 and 1 h Quantification is shown only for 0.5 h **(C)** mRNA expression of indicated genes in macrophages transfected with control and MFN2 siRNAs and treated with 100 ng/ml lipopolysaccharide (LPS) for 3 h **(D)** Western blot analysis and quantification of IKKβ phosphorylation in macrophages transfected with control and MFN2 siRNAs and stimulated with 100 ng/ml lipopolysaccharide (LPS) for 30 min. All results are shown as means ± SEM of at least three independent experiments compared using one-way analysis of variance (ANOVA) with Sidak’s multiple comparisons test; *p < 0.05; **p < 0.01; ****p < 0.0001; ns, not significant.

Since TLR2 activation is also required for inflammatory cytokine production in zymosan-simulated murine macrophages (46), we evaluated the impact of MFN2 on TLR2-dependent signaling. In these experiments we used TLR2/TLR6 agonist Pam2CSK4 (PAM2) to stimulate control and MFN2-silenced human primary macrophages, analyzing cytokine gene expression as well as activation of NF-κB and MAPK signaling pathways. After 3 h incubation with 10 ng/ml PAM2 MFN2 knockdown macrophages showed significantly increased gene expression of pro-inflammatory cytokines, including *TNFα* and *IL6* (**Figure 4A**). At the same time gene expression of the anti-inflammatory cytokine *IL10* was decreased as compared to PAM2-stimulated, control siRNA-transfected macrophages (**Figure 4A**). Besides, MFN2 silencing enhanced PAM2-induced phosphorylation of IKKβ and p38 after 30 min and 1 h stimulation (**Figure 4B**). These data indicate that MFN2-inactivated human primary macrophages display elevation of TLR2-mediated inflammatory responses.

Having shown that MFN2 deficiency increases the activity of TLR2 signaling pathway, we also questioned the impact of MFN2 on signaling by TLR4 in macrophages stimulated with LPS. In contrast to the murine MFN2-knockout macrophages, which exhibited diminished responses to LPS (30), MFN2 silencing increased TLR4-mediated signaling and cytokine expression, although the effect was less pronounced as compared to zymosan-induced inflammatory response (**Figures 4C, D**).

**Figure 5:**
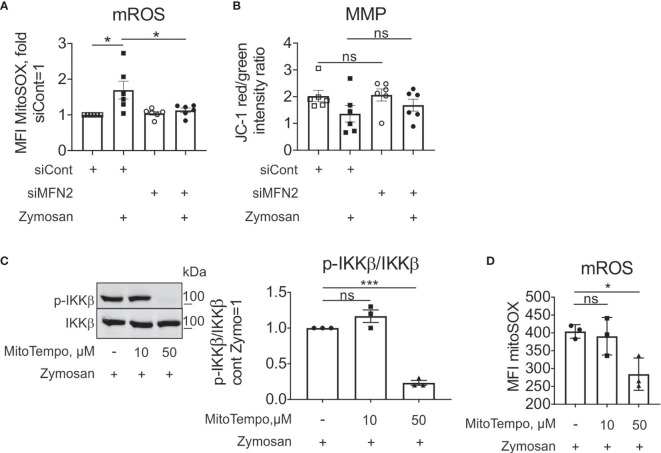
MFN2 impact on zymosan-stimulated signaling is mROS-independent. **(A)** mROS production in control and MFN2-silenced macrophages was measured by MitoSOX staining. Cells were treated with 50 µg/ml zymosan for 30 min. **(B)** Mitochondrial membrane potential (MMP) in control and MFN2-silenced macrophages was measured by JC-1 staining. Cells were treated with 50 µg/ml zymosan for 30 min. **(C)** Western blot analysis and quantification of IKKβ phosphorylation in macrophages pre-incubated for 30 min with mROS inhibitor MitoTEMPO (10 or 50 µM) followed by zymosan (50 µg/ml) stimulation for 30 min. **(D)** mROS production in macrophages pre-incubated for 30 min with MitoTEMPO (10 or 50 µM) prior to zymosan (50 µg/ml) stimulation for 30 min. All results are shown as means ± SEM of at least three independent experiments compared using one-way analysis of variance (ANOVA) with Sidak’s multiple comparisons test; *p < 0.05; ***p < 0.001; ns, not significant.

We proceeded to investigate mechanisms how MFN2 deficiency impacts zymosan-stimulated signaling. ROS are essential in regulating protein kinases activation, gene expression (50), calcium channels and transporters function (51), causing oxidative damage to the body (52). Mitochondria are the main source of ROS production in most cells (53, 54). We measured mROS using mitochondria-specific red fluorescent ROS sensor MitoSOX. Our previous observations validated mitochondrial location of the MitoSOX signal in human macrophages (34). Zymosan increased mROS levels in cells transfected with control siRNA (**Figure 5A**). MFN2 silencing did not alter mROS production under basal conditions, but decreased mROS levels in zymosan-stimulated macrophages (**Figure 5A**). This contrasts observations in murine macrophages, where MFN2 deficiency caused a substantial decrease of mROS production under resting conditions (30). Macrophages with a MFN1 knockdown still produced increased levels of mROS upon zymosan stimulation (**Supplementary Figure 2**). Whereas in the murine system decreased mROS generation in MFN2 knockout macrophages was accompanied by diminished mitochondrial membrane potential (MMP), we did not observe any change in MMP in MFN2-silenced macrophages under basal conditions as well as after zymosan stimulation (**Figure 5B**).

To test the impact of mROS on zymosan-stimulated signaling in our experimental system, we used a specific scavenger of mitochondrial superoxide MitoTEMPO, and evaluated zymosan-induced IKKβ activation and mROS levels. We pre-incubated macrophages for 30 min with 10 and 50 µM MitoTEMPO followed by zymosan treatment for 30 min. 50 µM MitoTEMPO blocked zymosan-induced IKKβ phosphorylation (**Figure 5C**) and significantly inhibited mROS production in zymosan-stimulated macrophages (**Figure 5D**). These data suggest that mROS support zymosan-induced signaling. As MFN2 deficiency is characterized by decreased mROS upon zymosan stimulation, but increased activation of IKK, the stimulatory effect of MFN2 loss is likely through ROS-independent pathways.

**Figure 6:**
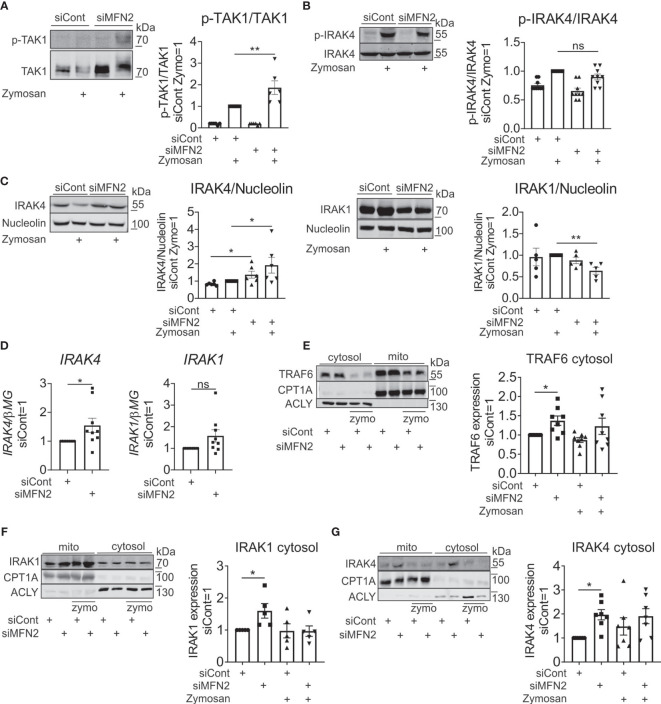
MFN2 affects NF-κB signaling through cytosolic expression of IRAK4 signaling complex. **(A)** Western blot analysis and quantification of TAK1 phosphorylation in control and MFN2-silenced macrophages stimulated with zymosan (50 µg/ml) for 30 min. **(B)** Western blot analysis and quantification of IRAK4 phosphorylation in control and MFN2-silenced macrophages stimulated with zymosan (50 µg/ml) for 30 min. **(C)** Western blot analysis and quantification of IRAK4 and IRAK1 in control and MFN2-silenced macrophages stimulated with zymosan (50 µg/ml) for 30 min. **(D)** mRNA expression of IRAK4 and IRAK1 in control and MFN2-silenced macrophages. **(E–G)** Western blot analysis and quantification of TRAF6 **(E)**, IRAK1 **(F)**, and IRAK4 **(G)** proteins in cytosolic and mitochondrial fractions from zymosan-treated control and MFN2-silenced macrophages. All results are shown as means ± SEM of at least three independent experiments compared using one-way analysis of variance (ANOVA) with Sidak’s multiple comparisons test or Student unpaired, two-tailed t-test; *p < 0.05; **p < 0.01; ns, not significant.

To further explore the role of MFN2 in zymosan-induced inflammatory response in human primary macrophages, we evaluated upstream kinases of NF-κB signaling pathway. We examined activation of transforming growth factor beta-activated kinase 1 (TAK1), which is linked to IL-1 and TNFα receptor signaling and activated by TLR ligands (55, 56). TAK1 together with TAK1 binding proteins activate two different pathways, IKK complex and the MAPK pathway (57, 58). Phosphorylation of TAK1 was significantly increased in MFN2-silenced macrophages (**Figure 6A**). Next, we explored whether activation of inflammatory response by MFN2 knockdown in zymosan-stimulated macrophages occurs upstream of TAK1 activation at MyD88-IRAK signaling complex (59). In TLR signaling pathways adaptor MyD88 recruits IRAK1 and IRAK4, which are activated by phosphorylation. Then IRAK associates with ubiquitin ligase TNF receptor associated factor 6 (TRAF6), causing activation of MAPK and NF-κB signaling pathways (60). Therefore, we tested if MFN2 deficiency affects IRAK4 or IRAK1 activation. A MFN2 knockdown altered phosphorylation of IRAK4 neither after zymosan treatment, nor after TLR2 agonist PAM2 (**Figure 6B** and **Supplementary Figure 3A**). However, IRAK4 protein levels increased in MFN2-inactivated macrophages, basally as well as after zymosan stimulation (**Figure 6C**). Additionally, we tested IRAK4 mRNA expression, which was also enhanced after MFN2 silencing (**Figure 6D**). We did not observe any change in protein level or gene expression of IRAK1 in MFN2-deficient cells at the basal level, although zymosan decreased IRAK1 protein after the MFN2 knockdown, which may reflect enhanced protein ubiquitination and degradation (61) (**Figures 6C, D**). Furthermore, expression of TLR2 mRNA remained unaltered in MFN2-silenced macrophages whereas mRNA levels of the TLR2 heterodimeric partner TLR6 were reduced after MFN2 knockdown (**Supplementary Figure 3B**). These findings demonstrate that MFN2 silencing may increase signal transduction *via* increased IRAK4 expression without affecting its activation.

Cytosolic proteins that interact transiently with TLR signaling complexes can translocate to mitochondria, as shown for TRAF6 (62), MyD88, IRAK4, and IRAK2 (63). We hypothesized that in our system similar translocations can take place. In this regard we performed cellular fractionation and mitochondrial isolation in order to compare levels of TRAF6, IRAK4 and IRAK1 in cytosolic and mitochondrial fractions. We found substantial amounts of these proteins in the mitochondrial fraction of unstimulated macrophages, which was not further enhanced upon zymosan stimulation. TRAF6 expression was increased in the cytosolic fraction after MFN2 silencing at the basal level (**Figure 6E**). Protein levels of IRAK4 and IRAK1 were also elevated in cytosol in MFN2-inactivated macrophages at the basal level (**Figures 6F, G**). At the same time, mitochondrial levels of these proteins remained unaltered in MFN2-silenced cells (**Supplementary Figure 3C**). These results indicate that the MFN2 knockdown does not affect the redistribution of TRAF6/IRAK1/IRAK4 complexes between cytosol and mitochondria. However, we observed increases of total and cytosolic levels of IRAK4 in MFN2-silenced macrophages. These findings suggest that MFN2 affects NF-κB signaling through cytosolic expression of IRAK4 signaling complex.

## Discussion

In the present study, we explored the role of MFN2 in mitochondrial function and inflammatory responses of primary human macrophages. The phenotype of MFN2 knockout macrophages is well described in the mouse (30), however there is little information towards human macrophages.

MFN2 is a mitochondrial outer membrane GTPase, which regulates mitochondrial fusion, and sustains mitochondrial homeostasis (9). Consistent with the role of MFN2 in fusion of outer mitochondrial membranes, MFN2 silencing in human primary macrophages resulted in minor fragmentation of mitochondrial networks. These findings support observations in MFN2-deficient mouse embryonic fibroblasts (64), macrophages (30), and cardiomyocytes (65), which demonstrate fragmentation of mitochondria. However, the fragmentation phenotype was mild as compared e.g. to more pronounced disruption of mitochondrial networks induced by fatty acids in human macrophages (34). It should be noted that although both MFN1 and MFN2 promote mitochondrial fusion, absence of MFN1 in embryonic fibroblasts causes severe mitochondrial fragmentation with formation of small spheres, while MFN2 deficiency causes only mild fragmentation (64). Furthermore, overexpressed MFN1 in HeLa cells tethers mitochondria more effectively than MFN2, correlating with a higher GTPase activity of MFN1 (66). In human MFN2-deficient macrophages we see small round mitochondria as well as normal mitochondrial networks. This partial fragmentation may indicate that compensatory mechanisms operate. Indeed, our observations of increased MFN1 expression in MFN2-silenced macrophages support the notion that MFN1 elicits compensatory up-regulation in MFN2-silenced macrophages. Thus, MFN1 functionally balances mitochondrial fusion defects caused by the absence of MFN2 (66, 67). We also note that MFN2-deficient murine macrophages appear to have a more severe fragmentation phenotype (30). It is unclear whether this is due to the complete absence of MFN2 or the lack of MFN1 up-regulation in the murine system. At the same time, the total amount of mitochondria was not affected by the MFN2 knockdown in human primary macrophages, as reported for cardiac myocytes (68) and for mouse macrophages (30).

Cells with impaired mitochondrial fusion have defects in respiratory function (69). In cardiomyocytes from MFN2 conditional knockout mice and in MFN2-ablated MEFs reduced mitochondrial respiration was observed (65). Similarly, MFN2-deficient mouse macrophages show significantly attenuated spare respiratory capacity, but their basal and ATP-coupled respiration as well as glycolysis remained unaltered (30). In human macrophages the MFN2 knockdown reduced basal as well as maximal OCR, but also attenuated ECAR, reflecting an overall decreased metabolic activity of MFN2-silenced macrophages. Surprisingly, this occurs without any loss of MMP, which is in contrast to a significantly reduced MMP in MFN2-deficient murine macrophages (30). Collectively, MFN2 silencing in human macrophages attenuates mitochondrial networking and mitochondrial metabolism, but the mitochondrial phenotype is less severe in comparison to MFN2 knockout mouse macrophages.

Besides its role in controlling mitochondrial morphology, MFN2 is involved in interactions between ER and mitochondria. The close contacts between ER and mitochondria, also known as mitochondria-associated membranes, consist of various proteins with different functions and play crucial roles in calcium exchange, lipid biosynthesis, cell survival, and immune signaling (70–72). In mammalian cells MFN2, located not only on the outer mitochondrial membrane, but also on the ER surface (13), was characterized as a protein controlling tethering of mitochondria and ER (11, 13, 73). In line, our findings in human macrophages show reduction of ER-mitochondrial contacts after MFN2 knockdown.

MFN2-deficient murine macrophages show impaired inflammatory responses to bacterial lipopolysaccharide (30). Unexpectedly, when assessing the impact of MFN2 silencing on the response of human macrophages to zymosan, yeast cell wall particles, we observed an increased production of inflammatory mediators. This included elevated expression and secretion of IL1β, TNFα, IL6 and IL8, as well as increased COX2 protein levels and PGE_2_ synthesis. Furthermore, the MFN2 loss increased zymosan-induced NF-κB and MAPK inflammatory signal transduction in human primary macrophages. This differs from results obtained in LPS-stimulated mouse macrophages, where a MFN2 knockout reduced pro-inflammatory cytokine production as well as p38 and ERK phosphorylation (30). Tur et al. casually linked this effect to the decrease of mitochondrial and total ROS levels (30). Whereas in our system no change in mitochondrial or total ROS production was noticed at basal level, we detected less mROS upon zymosan stimulation in MFN2 knockdown macrophages. Experiments with a specific scavenger of mitochondrial superoxide MitoTEMPO confirmed that mROS support pro-inflammatory signaling in zymosan-stimulated macrophages. Despite reduced mROS, MFN2 silencing enhances activation of NF-κB and MAPK cascades in our system. This suggests that the pro-inflammatory effect of MFN2 deficiency overrides the anti-inflammatory action of suppressed mROS levels.

Mannan and β-glucan components of zymosan are recognized mainly by dectin-1 and TLRs (46, 47). Thus, in rat alveolar macrophages and RAW 264.7 cells zymosan binding to TLR2 and TLR6 receptors underlies activation of NF-κB and production of TNFα (35, 47, 74). Collaboration of dectin-1 with TLR2 is required to produce inflammatory cytokines and induce antimicrobial ROS responses (46). In primary human monocytes dectin-1 was identified as a major receptor that mediates zymosan activation of NADPH oxidase and the phagocytic process (49). Our findings in human primary macrophages suggest that dectin-1 plays minor roles in zymosan-induced inflammatory responses and this signaling pathway is not affected by MFN2 knockdown. Surprisingly, we did not see activation of Syk or Src tyrosine kinases, major mediators of dectin-1 signaling, after zymosan stimulation. We also did not detect any differences in total ROS production or phagocytosis of fluorescently labeled zymosan particles between zymosan-stimulated control and MFN2-silenced primary macrophages. Interestingly, Src phosphorylation was increased in unstimulated, MFN2-silenced macrophages. The mechanism and implications of this elevation for macrophage physiology requires further investigation. Not observing an impact of the Src inhibitor on NF-κB activation suggests that in our system Src does not significantly contribute to zymosan-stimulated pro-inflammatory signaling.

In contrast, experiments with the TLR2/6 agonist Pam2CSK4 (PAM2) showed elevation of TNFα and IL6 expression and increased NF-κB and MAPK inflammatory signal transduction after MFN2 knockdown in PAM2-stimulated macrophages. Moreover, we detected activation of TAK1, an upstream kinase of NF-κB signaling pathway, in zymosan-induced, MFN2-inactivated human primary macrophages. These observations support that MFN2 induces TLR2-mediated inflammatory responses in human primary macrophages. We also observed elevation of TLR4-mediated cytokine expression and NF-κB activation after MFN2 depletion, although this was less pronounced as compared to MFN2 knockdown effects in zymosan-stimulated cells. This again underscores the differences in the outcome of MFN2 inactivation between human and murine macrophages.

Our data indicated that the effect of MFN2 occurs upstream of the TAK1/IKK node in the TLR2 signaling cascade. The MyD88-dependent pathway is common for all TLRs (59). In signaling pathways *via* TLRs adaptor MyD88 recruits IRAK1 and IRAK4, which are activated by phosphorylation and ubiquitination. The activated complex associates with TRAF6, causing activation of downstream MAPK and NF-κB signaling (60). Phosphorylation of IRAK4 was altered in MFN2-defficient human macrophages neither after zymosan treatment, nor after PAM2, suggesting that upstream events leading to IRAK4 activation are not affected by MFN2. However, protein and mRNA levels of IRAK4 were elevated in MFN2-inactivated human macrophages at the basal level as well as after zymosan stimulation.

West et al. demonstrated that TLR1, TLR2, and TLR4 are involved in the recruitment of mitochondria to phagosomes in murine RAW 264.7 macrophages (62). Cytosolic proteins can interact with TLR signaling complexes and translocate to mitochondria (62, 63). For example, TRAF6 relocates to mitochondria where it ubiquitinates evolutionarily conserved signaling intermediate in Toll pathways protein, resulting in elevated levels of mitochondrial and cellular ROS (62). However, MyD88, IRAK4, IRAK1, TAK1, and IκBα were not detected in mitochondrial fractions (62). In primary mouse adipocytes MyD88, IRAK4 and IRAK2, but not IRAK1, translocate to mitochondria upon IL-1β stimulation (63). In human macrophages we detected TRAF6, IRAK1 and IRAK4 in the mitochondrial fraction of unstimulated macrophages, and stimulation with zymosan did not alter their levels. MFN2 silencing did not redistribute TRAF6, IRAK1 or IRAK4 from cytosol to mitochondria. However, basal expression of these proteins was increased in cytosolic fraction after MFN2 silencing.

Collectively, these findings provide insights into potential roles of MFN2 in inflammatory responses in human primary macrophages, discovering major differences between murine and human systems regarding the impact of MFN2 on mitochondrial function and inflammatory signal transduction. We speculate that MFN2 affects NF-κB inflammatory signal transduction by altering the cytosolic expression of IRAK4 signaling complex, which is not associated with mROS production. Further work should elucidate the mechanisms how MFN2 regulates IRAK4 expression.

## Data Availability Statement

The raw data supporting the conclusions of this article will be made available by the authors, without undue reservation.

## Author Contributions

VK conceived and performed the experiments, analyzed the data, and drafted the manuscript. YS, GG, and RB contributed to data analysis and interpretation. DN and BB participated in study design, data analysis, and writing of the final manuscript draft. All authors contributed to the article and approved the submitted version.

## Funding

This study was supported by the grants from Deutsche Forschungsgemeinschaft (SFB 1039, Teilprojekt A01, A05, B04, and Z01).

## Conflict of Interest

The authors declare that the research was conducted in the absence of any commercial or financial relationships that could be construed as a potential conflict of interest.

## Publisher’s Note

All claims expressed in this article are solely those of the authors and do not necessarily represent those of their affiliated organizations, or those of the publisher, the editors and the reviewers. Any product that may be evaluated in this article, or claim that may be made by its manufacturer, is not guaranteed or endorsed by the publisher.
