# Supplementary material for: Mitofusin 2 Deficiency Causes Pro-Inflammatory Effects in Human Primary Macrophages
Source: Front Immunol. 2021 Aug 12;12:723683. doi: 10.3389/fimmu.2021.723683 (PMC8397414; doi:10.3389/fimmu.2021.723683)
Supplement: Supplementary file 1 [file DataSheet_1.pdf]

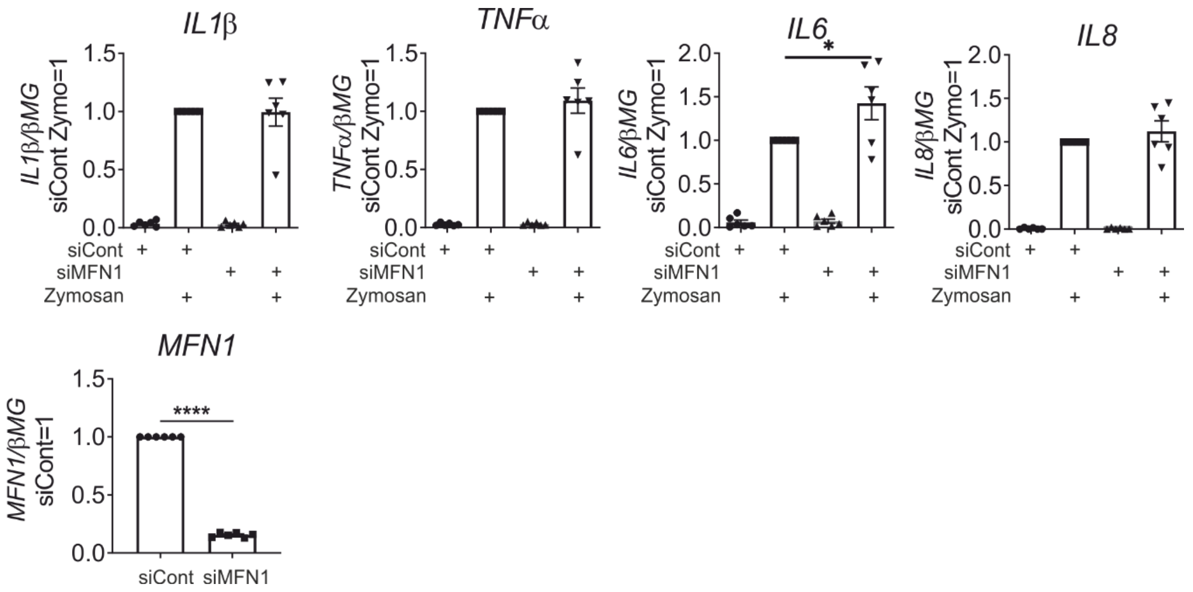

Supplementary Figure 1.
mRNA expression of indicated genes in macrophages transfected with control and MFN1 siRNAs and treated with 50 µg/ml zymosan for 3 h.

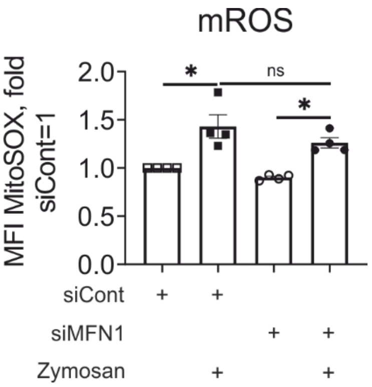

Supplementary Figure 2.
mROS levels in macrophages transfected with control and MFN1 siRNAs and treated with 50 µg/ml zymosan for 30 min.

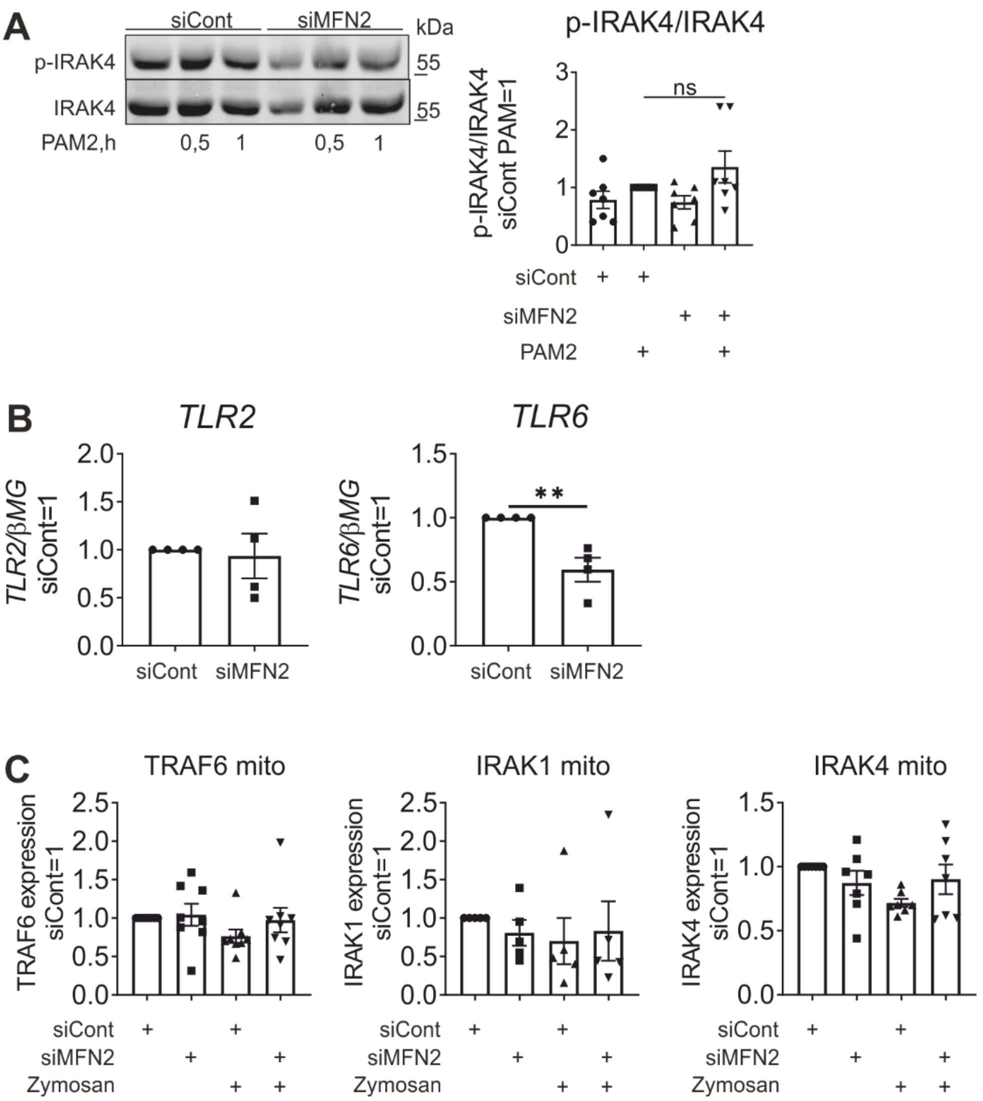

Supplementary Figure 3.

A, Western blot analysis and quantification of IRAK4 phosphorylation in control and MFN2-silenced macrophages stimulated with PAM2CSK4 for 30 and 60 min. B, mRNA expression of TLR2 and TLR6 in macrophages transfected with control and MFN2 siRNAs. C, quantification of TRAF6, IRAK1, and IRAK4 proteins in mitochondrial fractions from zymosan-treated control and MFN2-silenced macrophages.
